# Burnout among midwives*—*the factorial structure of the burnout assessment tool and an assessment of burnout levels in a Swedish national sample

**DOI:** 10.1186/s12913-022-08552-8

**Published:** 2022-09-16

**Authors:** Emina Hadžibajramović, Malin Hansson, Magnus Akerstrom, Anna Dencker, Gunnel Hensing

**Affiliations:** 1 Institute of Stress Medicine, Region Västra Götaland, Gothenburg, Sweden; 2 School of Public Health and Community Medicine, Institute of Medicine, Sahlgrenska Academy at the University of Gothenburg, Gothenburg, Sweden; 3 Institute of Health and Care Sciences, Sahlgrenska Academy, University of Gothenburg, Gothenburg, Sweden

**Keywords:** Burnout Assessment Tool, BAT, Midwives, Burnout, Rasch analysis

## Abstract

**Background:**

Many workplaces, within the healthcare sector, experience high rates of mental health problems such as burnout, anxiety, and depression, due to poor psychosocial working conditions and midwives are not an exception. To develop preventive interventions, epidemiologic surveillance of burnout levels, and their relation to professional specific working conditions is needed. Aims of this study is to assess the construct validity of the Burnout Assessment Tool (BAT) in the context of Swedish midwives, to evaluate whether the item responses can be combined into a single score and differential item functioning regarding age. Another aim was to assess the burnout levels of Swedish midwives.

**Methods:**

Data come from a national cohort of Swedish midwives (*n* = 1664). The construct validity was evaluated using Rasch analysis. Burnout levels were presented by median and first (Q1) and third (Q3) quartiles for the BAT total score and the four subscales (exhaustion, mental distance, cognitive and emotional impairment).

**Results:**

In the analysis including all 23 items the fit to the Rasch model was not obtained. Items within each subscale clustered together in a residual correlation matrix in a pattern consistent with the underlying conceptualization of the BAT, indicating multidimensionality. The Rasch analysis was re-run using the four testlets as input variables which resulted in a good fit. The median burnout level was 2.0 (Q1 = 1.6, Q3 = 2.4). The four subscales differentiated the picture (elevated levels on exhaustion and low levels on the other three subscales).

**Conclusions:**

The construct validity of the BAT for use in the context of Swedish midwives was confirmed. The results indicated a strong general factor, meaning that the responses can be combined into a single burnout score. The scale works invariantly for different age groups. The results of this study secure access to a validated instrument to be used for accurate assessment of the burnout levels among midwives in Sweden.

**Supplementary Information:**

The online version contains supplementary material available at 10.1186/s12913-022-08552-8.

## Background

Many workplaces, in particular within the healthcare sector, experience high rates of mental health problems such as burnout, anxiety, and depression, due to poor psychosocial working conditions such as high job demands, low job control and low social support [1–3]. Midwives and their working conditions are of course not an exception. When investigating the work-related health of midwives, previous studies have demonstrated high levels of burnout [4, 5]. Consequently, these high levels of burnout among midwives have a large effect on both the individuals themselves [6] and the organisations, in terms of reduced quality of care [7, 8], high sickness absence rates [9], and increased employee turnover for midwives, resulting in a midwifery shortage [10, 11]. In Sweden, acute and prolonged reactions to stress are the largest diagnostic groups in sickness absence, and employees in female dominated sectors are worst affected [12]. Swedish midwives report the third-highest level of sick leave among health-care professionals [13]. Maternity care must always maintain a functioning service and cannot postpone or delay care. To develop preventive interventions, epidemiologic surveillance of burnout levels, and their relation to professional specific working conditions is needed. Thus, valid and reliable instruments to assess employee burnout levels are needed.

Several methodological challenges have been presented in the assessment of burnout. For a start various burnout questionnaire exist e.g. the Copenhagen Burnout Inventory [14], the Shirom Melamed Burnout Questionnaire [15], the Maslach Burnout Inventory (MBI) [16] as well as many others and many of them have different conceptualisations of burnout. Moreover, different assessment tools have been used in different countries and studies which do not enable comparisons between these [17]. In addition, the assessment tool that has been used most frequently in research, the Maslach Burnout Inventory (MBI) [16] has been criticized on both theoretical as well as empirical grounds [18–24]. To overcome these shortcomings, the Burnout Assessment Tool – BAT was developed [25, 26].

According to the BAT, burnout is defined as a work-related state of exhaustion among employees, characterized by extreme tiredness, reduced ability to regulate cognitive and emotional processes, and mental distancing [26]. The BAT’s definition of burnout is built on the analysis of Schaufeli and Taris [18], where burnout is described as the combination of the inability and the unwillingness to no longer expend necessary effort at work and is reflected by its energetic and motivational component respectively. The inability is manifested by the lack of energy. The unwillingness to spend effort is characterized by increased resistance, reduced commitment, lack of interest, disengagement (i.e. mental distancing). According to Schaufeli et al. [26] there are four core symptoms of burnout (exhaustion, mental distance, emotional and cognitive impairment) as well as three secondary symptoms (depressed mood, psychological distress and psychosomatic complaints).

At present, the BAT has shown a high validity and reliability, and sound psychometric properties in the initial validation studies [26–28] but there is a need for further validation studies for the use in different countries and contexts. This is the first study of levels of burnout, using the BAT, both in a Swedish context and for midwives. The validation studies can be conducted in different ways addressing different aspects of validity. The Rasch measurement model [29], usually referred to as Rasch analysis, belongs to the modern psychometric approaches or item response theory (IRT) and is often used to evaluate construct validity of instruments. According to Pendrill [30] the Rasch model is a specific metrological approach to human-based measurements. A comprehensive overview of the statistical theory of Rasch models is given in a textbook edited by Christensen et al. [31] and a short introduction can be found in many articles [32–34].

The objective of this study was to assess the construct validity of the Swedish version of BAT in the context of Swedish midwives, using Rasch analysis. More specifically, the goal is to evaluate whether the responses on the four subscales can be combined into a single burnout score as well as possible differential item functioning regarding age. A second objective (subject to the condition that the first objective was fulfilled) is to assess the burnout levels in a national sample of Swedish midwives.

## Methods

### Study population

In 2020 a web-survey was distributed to all Swedish midwives who were members of either of two unions: the Swedish Association of Midwives or the Swedish Association of Health Professionals. These two unions organize the majority of the midwives in Sweden. An invitation to participate was sent out to 5,076 midwives and 2,060 choose to participate, generating a participation rate of 41%. Of the participating midwives 99.6% were women with a mean age of 48 years. The gender and age distribution of the participants was in line with public national statistics on midwives. The workplaces of midwives in this study included labour ward (44%), maternity care (32%), postnatal care (29%), gynaecology (11%) and youth clinic (8%).

The Swedish Ethical Review Authority approved the study in 2019 (Dnr 2019–03,776) and it was performed in accordance with the Declaration of Helsinki from 1964 and its later amendments regarding research involving human subjects. All participants were assured of confidentiality. The participants gave informed consent before taking part in the study.

### Data collection

A professional data collection company generated individual links to the web survey. These links were distributed by the two unions to all midwives with registered e-mail addresses. Three reminders were sent out. It was made clear in the information to the participants that the unions were not involved in any way in the study, other than distributing the invitation and reminders with individual links to the web-survey. Collection of data began on 04/02/2020 and ended on 20/04/2020. Note that the data collection was done before the full effect of the Covid-19 pandemic hit Sweden. A database was created by the data collection company and handed over to the researchers for analysis.

### The burnout assessment tool (BAT)

The Burnout Assessment Tool (BAT) is a self-report questionnaire consisting of 23 items (see Supplementary Information – (Supplementary file 1) for the Swedish version used in this study) grouped in four subscales: exhaustion (EX, 8 items), mental distance (MD, 5 items), cognitive impairment (CI, 5 items), and emotional impairment (EI, 5 items). All items are expressed as statements with five frequency-based response categories (1 = never, 2 = rarely, 3 = sometimes, 4 = often, 5 = always). The total burnout score, as well as scores for each subscale is calculated as a mean value of all responses, where a high score is indicative of high level of burnout (range 1–5). In the present study Swedish version of the BAT was used. The translation of the BAT to Swedish was done using both the original (Dutch version) and the English version of the instrument by means of dual-panel translation including the bilingual panel and the lay panel. For the English-Swedish translation, the panel consisted of a professional translator, trained clinicians with a long experience of treating patients with stress-related problems as well as researchers in the field, all fluent in both Swedish and English. For the Dutch-Swedish translation the panel included a trained physician who is fluent on all three languages. In the first step all involved worked with their own translations. In the second step the panel members worked together in a series of meetings and had opportunity to discuss alternatives until they agreed on the most appropriate and conceptually equivalent translation. In the final step, a lay panel consisting of a small group of employees in Region Västra Götaland, tested a Swedish version of the BAT and had the opportunity to give their comments. The translation process took place at the Institute of Stress Medicine in Gothenburg, Sweden.

### The Rasch model

The goal of the Rasch analysis is to evaluate whether the observed data satisfy the requirements of the Rasch model, in which case the measurement shows construct validity, and the total score is a sufficient statistic for the latent construct that is being measured by the questionnaire. Important requirements in Rasch analysis are unidimensionality, monotonicity, invariance, absence of differential item functioning (DIF) and local independency, which are briefly explained here.

*Unidimensionality* is a basic assumption for combining a set of items into a single burnout score, i.e. all items should represent a common latent trait. *Monotonicity* implies that the item responses are positively related to the latent trait. The response structure required by the Rasch model is a stochastically consistent item order; i.e. a probabilistic Guttman pattern [35]. This implies that people experiencing higher levels of burnout are expected to get higher scores on the BAT and people with lower levels of burnout are expected to score lower on the BAT. Increasing levels of severity of burnout across response categories for each item need to be reflected in the data.

The assumption of *invariance* implies that the items need to work invariantly across the whole burnout continuum for all individuals, i.e. the ratio between the location values (positioning of items on the latent burnout logit scale) of any two items must be constant along the latent construct. Invariance also implies that the items need to work in the same way (invariantly) for all comparable groups, which is known as absence of *DIF*. Absence of DIF implies that given the same level of burnout, the scale should function in a similar way for comparable groups of respondents (e.g. women and men, younger and older).

Finally, *local dependency* implies that, having extracted the unidimensional latent trait of burnout, there should be no other meaningful patterns in the residuals. Assumption of local dependency may be violated by *response dependency* and/or *multidimensionality* [36]. Understandably, multidimensionality occurs when items are measuring more than one latent dimension. Response dependency occurs when items are linked in some way so that the response to one item depends on the response to another item. A pedagogical example from rheumatology is when several items assessing walking ability are included in the same questionnaire. If a person is able to walk several miles without difficulty, then that person is also able to walk 1 mile or less without difficulty [32]. Another form of response dependency, known as redundancy dependency, may be caused by the degree of overlap of the content of two items, so that a particular rating for one item logically implies the similar rating for another item, e.g. two items reflecting reversed statements such as “I feel tired” and “I feel alert” [37]. Local dependency affects the fit to the model. Response dependency can result in increased similarity of the responses of people across items, so that responses are more Guttman-like than they should be under no dependency. Contrarily, multidimensionality results would result in responses being less Guttman-like than they should be under no dependency [38].

### Data analyses

In the Rasch analyses, only complete cases on all study variables were considered. In total 2,060 respondents participated in the study, aged 25–70. Among these, excluded from the analyses were 344 participants who did not answer any of the BAT items, 20 participants who had missing values on one BAT item, one participant who had missing values on two BAT items and two participants who had missing values on three BAT items. Another 23 participants did not provide information regarding age and were excluded. Among remaining participants there were only three men and three who defined themselves as other gender. Therefore, it was not possible to evaluate DIF for gender and all analyses included only women. Thus, the final study sample comprised *n* = 1664 participants.

Rasch analysis incorporated a cross-validation strategy. This means that the data was split into two random samples and analysed twice, to check the robustness of the results. This is also a convenient way to adjust for possible problems associated with χ^2^ statistics in large sample sizes, where even minor levels of misfit become statistically significant. Rasch analysis was done in RUMM2030 [39]. Two random samples each consisting of 800 respondents were drawn using a function for random sampling in RUMM2030 and all analyses were done using these random samples The items’ fit was analysed using the partial credit model for polytomous cases [40]. To control for the large number of comparisons, the significance level was set at 0.01 and Bonferroni adjusted. The differences between the model-expected values and the observed values (i.e. residuals), are scrutinized in several ways in order to evaluate whether the data fit to the Rasch model.

In the first step, the first Rasch analysis included responses to all 23 items, to evaluate whether the items within each subscale would cluster together in a residual correlation matrix in a pattern that is consistent with the underlying conceptualization of the BAT. When instruments consist of a bundle of items measuring different aspects of the latent trait, it is expected that the correlation matrix of residuals reveals the clustering of the items within each subscale [37]. Any residual correlation between the items 0.2 above the average observed correlation is indicative of local dependency [37]. In the same analysis, the functioning of each item is evaluated in terms of (a) discriminant ability (item fit residual within range of ± 2.5); (b) threshold ordering (i.e., appropriateness of the response categories, evaluated graphically and by thresholds estimates for each item); (c) the non-significant item χ^2^ statistic; (d) local dependency (residual correlation matrix), and (e) absence of DIF for age (above/under the median age of 47). DIF was tested by conducting ANOVA of standardized residuals, which enables separate estimations of misfit along the latent trait, uniform and non-uniform DIF. Disordering of the thresholds was tested by the hybrid approach proposed by Salzberger [41].

The overall fit to the model was evaluated by the mean and standard deviations of person and item residuals (expected values are 0 and 1 respectively), the model χ2 statistic (expected to be non-significant and indicates invariance). The internal consistency of the scale and the power of the BAT scale to discriminate among respondents with different levels of burnout were evaluated with the Person Separation Index (PSI). The PSI ranges from 0 to 1 and is similar to Cronbach’s alpha.

Smith’s test of unidimensionality was used to investigate the assumption of unidimensionality [42]. For this test, first a principal component analysis (PCA) on residuals was performed. Items loading positively and negatively on the first principal component were used to obtain an independent person estimate. Finally, independent t-tests for differences in these estimates for each person were performed [42]. Less than 5% of such tests being outside the range of ± 1.96 support the unidimensionality of the scale. A 95% binomial confidence interval of proportions [43] was used to show whether the lower limit of the observed proportion is below the 5% level.

In the second step, (after the local dependency was confirmed in the first step) we followed the method of combining correlated items into testlets, as recommended by Marais et. al. and Andrich [36, 38, 44]. This method combines correlated items into one or more testlets (preferably based on theoretical considerations) and the data were re-analysed using testlets instead of individual items. Thus, the second model was run with the four testlets based on the BAT’s four subscales.

In the case of testlets, the total variance in the data can be divided into three components: a) the total common true score variance (across all items), b) the total unique variance (the sum of the unique variances among the subscales) and c) the total error variance of the scale. The testlets’ model fit was compared with the fit obtained from the initial analysis of the individual items [44]. The coefficient alpha from the initial analysis corresponds to the proportion of the non-error variance (the sum of a) and b) above) relative to the total variance and is compared to the coefficient alpha from the testlet analysis, which corresponds to the proportion of the common true score variance relative to the total variance. The proportion of the common true score variance to the non-error variance is also calculated as well as the average latent correlation among the subscales corrected for attenuation for error.

Finally, targeting (distribution on a logit scale) of the BAT items and persons in the sample was evaluated graphically in a person-item-threshold graph. Targeting evaluates how well the items are targeted for severity levels of burnout as reported by the respondents. For a well-targeted instrument, the mean location for persons would be around the value of zero. The person parameter estimates are compared with the distribution of the item thresholds. In that way, thresholds which are extreme compared to persons can be identified, as they provide little information in the population. This is important for the precision of person parameter estimates. In other words, responses to such items will have little impact on the precision of the person estimates as these items are out of target [45].

Burnout levels among midwives are presented in terms of median and first and third quartiles (Q1 and Q3 respectively) for the BAT total score (BAT) and well as for the four subscales (EX, MD, CI and EI). These measures were calculated in the total sample. The number of participants who completed all items (regardless of gender and age) was as follows: BAT *n* = 1,693, EX *n* = 1,711, MD *n* = 1,702, CI = 1,714 and EI *n* = 1,714. Differences in burnout levels between age groups were tested with the Mann–Whitney U test. The results are visualized with boxplots.

## Results

### Construct validity of the BAT in the context of Swedish midwives

The first analysis on the random sample 1, included all 23 items and the overall fit statistics are shown in Table 1. As expected, given the subscale structure of the BAT, fit to the model was not obtained. Item and person fit residuals mean and SD deviated from the expected value of 0 and 1 respectively and the value of χ2 statistic was high and significant (454.96, *p* < 0.0001). The value of PSI was high (0.94) but the Smith´s test indicated problems with dimensionality as the percentage of significant tests was 20.7 and the lower confidence bound was > 5%.

**Table 1:** Overall fit statistics in two random samples (*n* = 800 each) drawn from the Swedish Midwife Survey

	**Item residual**		**Person residual**		**Chi square**		**Unidimensionality**		
**Analysis name**	**Mean**	**SD**	**Mean**	**SD**	**Value**	**p**	**PSI**	**Test % (95% CI)**	**Alpha**
**Sample 1 *n* = 800**									
BAT 23 items	-0.22	2.94	-0.33	1.43	454.96	< 0.0001	0.94	20.73 (18.05;23.68)	0.94
BAT 4 testlets	-0.02	2.13	-0.34	0.87	44.53	0.16	0.84	5.40 (4.02;7.21)	0.81
**Sample 2 *n* = 800**									
BAT 23 items	-0.10	3.13	-0.34	1.45	489.15	< 0.0001	0.94	21.15 (18.46;24.12)	0.94
BAT 4 testlets	0.08	2.01	-0.36	0.93	35.34	0.50	0.85	4.88 (3.58;6.61)	0.81

Item fit statistics (residuals and thresholds) are shown in Table 2. As seen in Table 2, item fit residuals outside the predefined range of ± 2.5 were observed for several items.

**Table 2:** Item fit residuals, item thresholds and standard errors from the BAT23 analysis (bold indicates reversed thresholds), random sample 1 (*n* = 800) drawn from the Swedish Midwife Survey

		**Threshold (standard error)**			
**Item**	**Residual**	**1**	**2**	**3**	**4**
EX1	-2.74	-4.61 (0.081)	-2.21 (0.080)	-0.59 (0.111)	2.20 (0.335)
EX2	8.55	-5.32 (0.090)	-3.05 (0.078)	-1.36 (0.092)	0.75 (0.172)
EX3	-1.06	-5.00 (0.084)	-2.69 (0.078)	-0.53 (0.108)	1.00 (0.214)
EX4	-0.29	-3.63 (0.077)	-1.62 (0.087)	0.21 (0.142)	2.67 (0.495)
EX5	-1.53	-4.59 (0.080)	-2.25 (0.080)	-0.34 (0.117)	1.18 (0.239)
EX6	-4.87	-2.89 (0.076)	-0.84 (0.101)	0.69 (0.183)	3.24 (0.751)
EX7	-2.06	-3.34 (0.078)	-1.59 (0.089)	-0.14 (0.132)	1.62 (0.302)
EX8	-1.58	-5.22 (0.087)	-2.83 (0.078)	-1.13 (0.097)	0.95 (0.191)
MD1	-1.58	-3.77 (0.076)	-1.24 (0.091)	0.24 (0.150)	1.91 (0.377)
MD2	4.29	-2.51 (0.078)	-0.75 (0.105)	0.56 (0.181)	2.35 (0.509)
MD3	-0.03	-1.28 (0.086)	0.23 (0.148)	1.07 (0.274)	3.76 (1.184)
MD4	1.86	-1.12 (0.089)	0.04 (0.144)	1.04 (0.266)	1.86 (0.549)
MD5	2.90	-0.84 (0.094)	-0.02 (0.148)	1.04 (0.266)	3.00 (0.847)
CI1	-2.04	-3.91 (0.075)	-0.89 (0.095)	1.62 (0.245)	3.08 (0.907)
CI2	-3.39	-3.61 (0.075)	-0.92 (0.096)	1.77 (0.259)	4.23 (1.544)
CI3	-0.98	-3.79 (0.075)	-1.07 (0.093)	0.92 (0.186)	3.39 (0.833)
CI4	-2.99	-3.81 (0.076)	-0.75 (0.098)	1.41 (0.232)	4.43 (1.539)
CI5	1.30	-2.65 (0.079)	0.95 (0.158)	**3.71 (0.924)**	**1.99 (1.428)**
EI1	1.17	-2.22 (0.079)	0.58 (0.146)	3.46 (0.739)	5.85 (6.555)
EI2	-1.48	-1.57 (0.083)	0.10 (0.137)	1.79 (0.339)	2.82 (0.973)
EI3	1.87	-3.02 (0.076)	-0.78 (0.101)	1.03 (0.205)	7.68 (6.740)
EI4	-2.42	-1.34 (0.085)	0.37 (0.152)	1.42 (0.320)	6.96 (5.891)
EI5	2.07	-1.86 (0.080)	0.24 (0.137)	1.92 (0.363)	6.19 (4.523)

Evaluation of thresholds ordering showed that all items besides item CI5 had ordered thresholds. Item CI5 (*I make mistakes in my work because I have my mind on other things*) had a problem with thresholds three and four being reversed (Table 2) which correspond to the two highest response categories (*often* and *always*). Further analysis of the item CI5 was done using the Salzberger hybrid approach to evaluate true ordering (H_1_) or true disordering (H_2_) of thresholds three and four, with undecided results since the null hypotheses (no difference between the thresholds) could not be rejected (data not shown). Frequency distributions for all items show that the two highest response categories were used very rarely by the respondents in this study, see Supplementary file 2. Consequently, thresholds’ standard errors for these categories were much higher compared to the first two categories, especially for the item CI5 as well as for the EI items (Table 2). DIF for age was evaluated and no problems were observed for any of the items. A table showing the results from DIF analysis are given in Supplementary file 3.

The residual correlation matrix shown in Supplementary file 4 indicated problems with local dependency as correlations higher than expected under the condition of local independence (in this sample a value > 0.16) were found for many pairs of items. The pattern of the residual correlations corresponded to the underlying conceptual structure of the BAT with the four subscales, with high residual correlations found between pairs of items within the same subscales. The only exception was one pair of items between different subscales with an observed residual correlation of 0.19, namely items EX5 (*When I get up in the morning, I lack the energy to start a new day at work*) and MD1 (*I struggle to find any enthusiasm for my work*).

As a part of cross-validation strategy, the same analysis with all 23 items was repeated in random sample 2 and the results were similar. The overall fit statistics showed the same pattern as in the previous analysis (Table 1, Sample 2 BAT 23 items). For the item fit statistics (results not shown), significant item fit residuals outside the predefined range were found for the same two items, EX2 and MD2. Threshold disordering was observed for the item EI4 (*I get upset or sad at work without knowing why*) with two highest thresholds being reversed. The results of hypothesis testing for true ordering or true disordering were once again undecided. The pattern of residual correlations once again showed clusters of items within each subscale. The same pair of items (MD1 and EX5) was the only pair between the subscales with high observed residual correlation (0.20). No DIF for age was observed for any of the items. (Details about analyses for sample 2 can be obtained upon request from the first author.)

### The subscale structure of the BAT

In the next step, items within each subscale (exhaustion, mental distance, cognitive and emotional impairment) are combined into four testlets as a way of handling problems with local dependency between the items, and the Rasch analysis was re-run on the four testlets as input variables. This analysis resulted in a good fit to the Rasch model in both subsamples (Table 1, BAT 4 testlets analysis). Compared with the initial analysis, item and person residuals in the testlet analysis were closer to the expected values of 0 and 1. The value of the χ2 statistic dropped to 44.53 and 35.34 in the two samples respectively and was no longer significant. The percentage of the significant t-tests also dropped from 20.7% to 5.4% in sample 1 and the lower confidence bound was below the predefined value of 5%. Corresponding figures for sample 2 were 21.2% and 4.9% respectively.

As expected, due to the lower number of variables in the model, the value of PSI decreased to 0.84 and 0.85 in the two samples respectively. In the BAT23 analysis, the coefficient alpha, i.e. the proportion of the non-error variance out of the total variance in the data was 0.94 in both random samples. After accounting for local dependency by the testlet analysis, the value of alpha was 0.81, which corresponded to the proportion of the common true score variance (Table 1). In both random samples, the proportion of the common true score variance out of the non-error variance is 0.86, meaning that most of the non-error variance is explained by the true common score variance. Average latent correlation between the four subscales was 0.61. In all, these results justify that the 23 items can be combined into one measurement of total burnout*.* No DIF for age was observed.

**Fig. 1:**
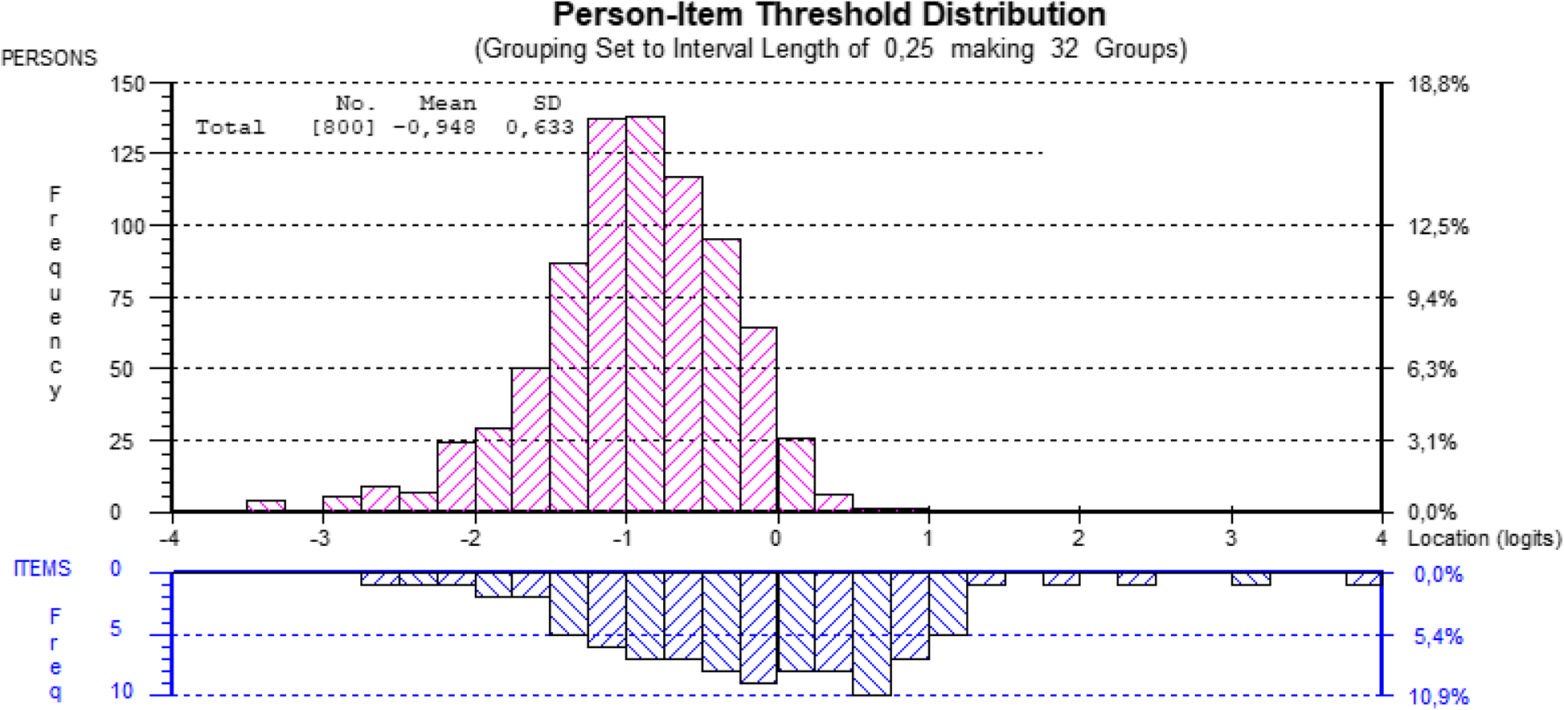
Person and item threshold distribution along the logit scale of burnout, based on the four testlets analysis (higher values indicate higher burnout levels) measured with the Burnout Assessment Tool on Swedish midwives

The targeting is visualized in Fig. 1 and shows the distribution of item thresholds (lower part of the figure) and persons (study participants) levels of burnout (upper part of the figure) along the common logit scale (higher values indicate higher burnout levels). The person mean was -0.95 (SD 0.63) on a logit scale and was lower than the items mean (constrained to 0). This means that rather low levels of burnout are observed in the sample, which was also seen in the frequency distribution of the items (Supplementary file 2). As explained above, the highest response categories were very rarely used by this occupation group.

### The burnout levels in Swedish midwives

Given the fit of data to the Rasch model, burnout levels in Swedish midwives were calculated. Frequency distributions for each item are shown in SI 2 (Supplementary file 2). Although the replies were distributed between all five response categories on all items, the frequencies of the two highest categories (often and always) were low on most of the items and especially for the items belonging to the MD, CI and EI subscales. Consequently, the first two response categories (never and seldom) were used by the majority of the respondents on these three subscales.

Median levels of burnout together with the first (Q1) and third (Q3) quartile for the overall burnout level (BAT-tot) as well as for the four subscales (EX, MD, CI, EI) for the total sample and for the two age groups (classified by the median age of 47) are presented in Table 3. The median burnout level for the total sample on the BAT-tot was 2.0. The Q1 was 1.6, meaning that the 25% of participants with the lowest scores rated their burnout levels in the range of 1 up to 1.6. In the same manner, 25% of the participants with the highest scores were observed in the range of 2.4 up to 4.4. The range for the total scores on each subscale for the BAT-tot are also shown in Table 3. Although there was a statistically significant difference between younger and older respondents in all five measures (*p* < 0.0001 for BAT-tot, EX, MD and CI and *p* = 0.006 for EI), their actual levels of burnout were comparable (Table 3).

**Table 3:** Burnout levels in Swedish midwives (*n* = 1664) measured with the Burnout Assessment Tool (BAT) (BAT-tot = total burnout score, EX = exhaustion, MD = mental distance, CI = cognitive impairment, EI = emotional impairment, Q1 = first quartile, Q3 = third quartile, younger = up to median age of 47, older = above median age)

	**BAT-tot**	**EX**	**MD**	**CI**	**EI**
**Median (Q1-Q3)**					
Younger	2.1 (1.7;2.5)	2.6 (2.1;3.1)	1.6 (1.2;2.2)	2.0 (1.6;2.6)	1.6 (1.2;2.0)
Older	1.9 (1.6;2.3)	2.4 (1.9;2.9)	1.4 (1.2;2.0)	2.0 (1.4;2.2)	1.4 (1.2;2.0)
Total sample	2.0 (1.6;2.4)	2.5 (2.0;3.0)	1.6 (1.2;2.0)	2.0 (1.6;2.4)	1.6 (1.2;2.0)
**Range (Min–Max)**					
Younger	1.0–4.4	1.0–5.0	1.0–4.4	1.0–5.0	1.0–4.6
Older	1.0–3.9	1.0–4.9	1.0–4.4	1.0–4.0	1.0–3.8
Total sample	1.0–4.4	1.0–5.0	1.0–4.4	1.0–5.0	1.0–4.6

**Fig. 2:**
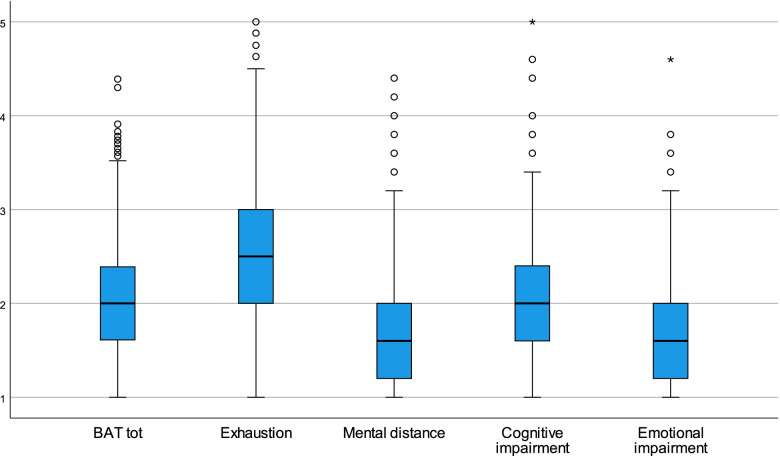
Score distributions of the BAT-total burnout and the four subscales measured with the Burnout Assessment tool on Swedish midwives

The scores of the four subscales can be used to differentiate the picture. The response profiles of midwives were visualised by boxplots in Fig. 2. Although the median level of BAT-tot was 2.0, average levels on the exhaustion scales were higher, where the value of 2.0 corresponded to the Q1. The opposite result was seen for the MD and EI, where the value of 2.0 corresponds to the Q3. The median value for the CI did not deviate from the median value of BAT-tot.

## Discussion

To the best of our knowledge, this is the first study to evaluate the construct validity of the Swedish version of the BAT, more specifically in the context of Swedish midwives. This was done by investigating whether the item responses of the four subscales can be combined into a single burnout score and whether the scale works invariantly between different age groups and was the first objective of the study. Validation of self-assessed instruments is an ongoing process, and questionnaires applied in new settings or new contexts call for new evaluations [46].

The results from the analyses of construct validity of the BAT in the context of Swedish midwives confirmed the theoretical foundation of the instrument, which defines the burnout as a syndrome, consisting of four inter-related symptoms and operationalised by the BAT’s four subscales. After accounting for the local dependency between the items within the four subscales, the data fit the Rasch model, meaning that the requirements for the scale to function as measurement of burnout are fulfilled. Thus, the responses of the items can be combined into a single burnout score. This measurement of burnout is invariant for age i.e. younger and older respondents use the scale in a comparable way given the same level of burnout.

Based on the view of burnout as a syndrome manifested by four inter-related symptoms, the results of the initial analysis with the 23 items not fitting the Rasch model and indicating multidimensionality was not surprising. Even if the results make perfect sense theoretically, local dependency is problematic from a measurement point of view and needs to be accounted in some way. In this study, we have accounted for local dependency by summarising the item responses within each subscale into testlets as recommended in the literature [36, 38, 44]. In the testlets analysis, the PSI value decreased from 0.94 to 0.84 and 0.85 in the two sample respectively but was still high enough to allow comparison of the BAT respondents with high precision. Corresponding figures for coefficient alpha were 0.94 (initial analysis) and 0.81 (testlet analysis).

The strength of our analysis is that we could combine the items into testlets on a theoretical basis. The testlet analysis confirmed a strong general factor meaning that the item responses can be combined into a single burnout score.

Our results showed that most of the systematic (non-error) variance in the data could be attributed to the common true score variance. The results are in line with a previous study by Hadzibajramovic et al. [28] where the construct validity of the original, Dutch version of the BAT was investigated using data from two representative sample of working populations in the Netherlands and Belgium (Flanders) and a study by de Beer et al. which investigated cross-national measurement invariance of the instrument [27]. In both studies, a strong general factor was demonstrated as well as in several other country-specific validation studies from different countries [47–51].

In the initial analysis including all 23 items, two item (CI5, *I make mistakes in my work because I have my mind on other things* in the first random sample and EI4, *I get upset or sad at work without knowing why*, in the second random sample) had disordered thresholds. For both items, the two highest categories (*often and always*) were reversed. Additional analyses were performed but the null hypothesis of no differences between the thresholds could not be ruled out, meaning that true disordering of the thresholds could not be concluded. One way to deal with disordered thresholds is to collapse response categories on that specific item and re-run the Rasch analysis. However, due to undecided results of the threshold ordering hypothesis and low frequencies on the two highest categories, which might be a possible explanation for estimates disordering, as well as the overall model fit problems, re-running the Rasch analysis with collapsed categories on item CI5 or EI4 was not done.

Furthermore, our results showed that the two highest categories (*often* and *always*) were very rarely used not only on CI5 and EI4 but on all other items as well for the subscales MD, CI and EI. One possible explanation may be response bias due to social desirability or acceptability of the responses. Participants might have avoided replying that they always or often make mistakes at work because they have their mind on other things. This social desirability bias might be stronger in a profession such as midwife since mistakes may have devastating effects. Another, more plausible explanation is that these responses are just not reasonable, given the tasks that midwives are expected to perform and in the context in which they work. We hypothesize that midwives who would have given these responses to a larger extent are unlikely to be able to perform their work tasks or maintain patient safety and are therefore on sick leave or have left the profession.

We also calculated and presented the burnout levels of the Swedish midwives which was the second objective of the study. Our results show that the median level of burnout in Swedish midwives is 2.0 on a scale ranging from 1 to 5. For interpretation of scores in terms of high and low burnout levels, the best option is to compare results with the statistical norms calculated on the national representative samples, as recommended in the BAT manual [25]. However, this is the first study of BAT in Sweden and thus, no representative Swedish sample is yet available. Comparing our observed median levels of burnout in Swedish midwives with the statistical norms from the Netherlands (NL) and Belgium (Flanders, FL) (women only), presented in the BAT manual, showed that the levels are approximately the same as in these two countries. However, the response profiles of midwives on the four subscales differ compared to NL and FL national representative levels. Compared to the NL and FL data (women only), Swedish midwives rated higher on the exhaustion subscale, lower on the mental distance and emotional impairment and had approximately the same levels of the cognitive impairment.

Based on these comparisons and previous research about the strained work situation of Swedish midwives with high demands and lack of organizational resources and support systems [52–54] it could be hypothesized that high levels of exhaustion in Swedish midwives are not surprising. Elevated level of exhaustion measured with the BAT in the present study, was also confirmed using a exhaustion subscale from the Copenhagen Psychosocial Questionnaire, an internationally recognized questionnaire developed to measure psychosocial work environment [55] in a study by Hansson, where it was concluded that a Swedish national sample of working midwives reported significantly higher exhaustion levels compared to Swedish benchmarks [52]. Moreover, rating low on the mental distance and emotional impairment items is also expected in relation to previous research where midwives reported a high meaningfulness in their work and in their professional role [52, 56]. Thus, based on the response profiles on the four subscales, we could say that the BAT is functioning as expected in the context of the Swedish midwives.

According to the definition of the burnout operationalized by the BAT, a possible evolution of the syndrome is from exhaustion to cognitive and emotional impairment to mental distance and back. Perhaps, in this data, we may possibly see early signals of burnout based on elevated exhaustion levels that partly may be mediated by the perceived high meaningfulness in their work and professional role, manifested as low levels of mental distance and emotional impairment. This reasoning is supported by Gregor et al. [57], stating that meaningfulness has been found to have a buffering role in relation to the work situations demands, and can, in addition, promote the motivational processes [57–59]. These findings are important, and actions need to be taken to reduce the high levels of exhaustion to prevent the situation from worsening, either by an even further increase in exhaustion or by increasing mental distance and emotional impairment due to the increased level of exhaustion. Such increases in the subscales would consequently lead to higher levels of burnout among Swedish midwives. To improve the adverse working conditions behind the elevated levels of exhaustion among Swedish midwives, measures with a good fit to the work environment challenge at hand need to be implemented [60, 61]. Designing such measures requires detailed information on the association between the levels of burnout and factors within the work environment which can only be derived by investigating the association between burnout levels and work environment factors, such as working condition, preferrable in a longitudinal study design, which is beyond the scope of this study.

An important strength of this first Swedish study on BAT is the nationwide sample of midwives which reduces effects of specific regions or hospitals. Further, the sample represents diversity in relation to workplaces. Earlier research mainly included midwives at labour wards or inpatient care. A limitation is that the comparisons are done with the statistical norms in NL and FL. However, so far there is no norm data from Sweden available. Few studies so far report the actual burnout levels within different occupational groups. Therefore, it is of course difficult to draw any firm conclusions and more studies are needed to be able to relate midwives’ levels of burnout and response profiles to other occupational groups, to Swedish statistical norms and to the short version of the BAT. The response rate in this study was 41%. It is possible that we could have observed other burnout levels if the response rate was higher. However, the distribution of age and gender of midwives in our sample is in line with the national statistics of midwives.

Lastly, practical implications of this study should be mentioned. One practical implication is that the results secure the access to a validated instrument that can be used by occupational health services in, for example, employee surveys, to accurately assess the levels of burnout among Swedish midwives and the relation of the burnout to factors within the work environment. Such knowledge may then be used to improve the working environment for midwives and to secure access to professionals within the maternity care. To support employers and researchers in interpreting the levels of burnout among midwives, we have calculated national representative norms for midwives. Thus, the burnout levels from this study can be used for comparisons within different work units.

## Conclusions

Using data from a representative sample of midwives in Sweden we have demonstrated that the Swedish version of the BAT can be used in the context of Swedish midwives. The instrument has good psychometric properties, and its construct validity is in line with the theoretical foundation of the instrument, in which burnout is seen as a syndrome with four inter-related symptoms, operationalized by the four subscales of the BAT. The item responses can be combined into a single burnout score and the scale works invariantly for younger and older midwives.

The median level of the midwife burnout was 2.0. The response profiles of the four subscales showed somewhat elevated level of exhaustion and relatively low levels on mental distance, cognitive and emotional impairment. Swedish national representative statistical norms are needed to relate their levels to the national level and to other occupations. The results of this study secure access to a validated instrument to be used for accurate assessment of the burnout levels among midwives in Sweden. Presented national representative burnout levels among midwives in Sweden can be used for comparisons at specific work units.

## Supplementary Information

**Additional file 1: Supplementary file 1.** Swedish version of the Burnout Assessment Tool.

**Additional file 2: Supplementary file 2.** Frequency distribution of the item responses on the Burnout Assessment Tool in Swedish Midwives.

**Additional file 3: Supplementary file 3.** Differential item functioning (DIF) for age.

**Additional file 4: Supplementary file 4.** Observed residual correlation matrix.

## Data Availability

The dataset generated and analysed during the current study is not publicly available as individual privacy could be compromised, but it is available from the corresponding author on reasonable request.

## Abbreviations

ANOVA: Analysis of variance
BAT: Burnout Assessment Tool
DIF: Differential item functioning
MBI: The Maslach Burnout Inventory
PCA: Principal component analysis
PSI: Person Separation Index
