# Supplementary material for: Burnout among midwives—the factorial structure of the burnout assessment tool and an assessment of burnout levels in a Swedish national sample
Source: BMC Health Serv Res. 2022 Sep 16;22:1167. doi: 10.1186/s12913-022-08552-8 (PMC9482233; doi:10.1186/s12913-022-08552-8)
Supplement: Supplementary file 1 — Additional file 1: Supplementary file 1. Swedish version of the Burnout Assessment Tool. [file 12913_2022_8552_MOESM1_ESM.pdf]

Supplementary file 1 – Swedish version of the Burnout Assessment Tool

Arbetsrelaterad version av BAT

Instruktion

Följande påståenden handlar om din arbetssituation och hur du upplever denna situation. Vänligen ange hur ofta varje påstående gäller för dig.

Skattning

Aldrig	Sällan	Ibland	Ofta	Alltid
1	2	3	4	5

Primära symptom

	Aldrig	Sällan	Ibland	Ofta	Alltid
Utmattning					
1. När jag är på arbetet känner jag mig psykiskt utmattad*	☐	☐	☐	☐	☐
2. Allting jag gör på arbetet kräver stor ansträngning	☐	☐	☐	☐	☐
3. Det är svårt för mig att återhämta mig efter en dag på arbetet*	☐	☐	☐	☐	☐
4. När jag är på arbetet känner jag mig fysiskt utmattad*	☐	☐	☐	☐	☐
5. Jag saknar energi för en ny dag på arbetet när jag går upp på morgonen	☐	☐	☐	☐	☐
6. Jag vill vara aktiv på arbetet men trots det klarar jag inte av det	☐	☐	☐	☐	☐
7. När jag anstränger mig på arbetet blir jag trött fortare än vanligt	☐	☐	☐	☐	☐
8. Vid arbetsdagens slut känner jag mig psykiskt utmattad och slutkörd	☐	☐	☐	☐	☐
Känslomässig distansering					
9. Jag får kämpa för att uppbringa entusiasm för mitt arbete*	☐	☐	☐	☐	☐
10. När jag är på arbetet går jag på autopilot och tänker inte på vad jag håller på med	☐	☐	☐	☐	☐
11. Jag känner stark motvilja för mitt arbete*	☐	☐	☐	☐	☐

Citation: Schaufeli, W.B., De Witte, H. & Desart, S. (2019). *Burnout Assessment Tool (BAT) – Test Manual.* KU Leuven, Belgium: Internal report.

12. Jag känner likgiltighet inför mitt arbete	☐	☐	☐	☐	☐
13. Jag känner mig cynisk inför vad mitt arbete har för betydelse för andra*	☐	☐	☐	☐	☐

	Aldrig	Sällan	Ibland	Ofta	Alltid
Kognitiv nedsättning					
14. På arbetet har jag svårt att vara fokuserad*	☐	☐	☐	☐	☐
15. På arbetet har jag svårt att tänka klart	☐	☐	☐	☐	☐
16. Jag är glömsk och distraherad på arbetet	☐	☐	☐	☐	☐
17. När jag arbetar har jag svårt att koncentrera mig*	☐	☐	☐	☐	☐
18. Jag gör misstag på arbetet eftersom jag tänker på annat*	☐	☐	☐	☐	☐
Nedsatt känsloreglering					
19. På arbetet känner jag mig oförmögen att kontrollera mina känslor*	☐	☐	☐	☐	☐
20. Jag känner inte igen mig själv i hur jag reagerar känslomässigt på arbetet*	☐	☐	☐	☐	☐
21. När jag arbetar blir jag lätt irriterad när saker inte går som jag vill	☐	☐	☐	☐	☐
22. Jag blir upprörd eller ledsen på arbetet utan att veta varför	☐	☐	☐	☐	☐
23. På arbetet händer det att jag överreagerar utan att mena det*	☐	☐	☐	☐	☐

Observera: * = Kort version

Sekundära symptom

	Aldrig	Sällan	Ibland	Ofta	Alltid
Psykisk påfrestning					
1. Jag har svårt att somna eller fortsätta sova när jag väl har somnat	☐	☐	☐	☐	☐
2. Jag oroar mig lätt	☐	☐	☐	☐	☐
3. Jag känner mig spänd och stressad	☐	☐	☐	☐	☐
4. Jag känner mig ängslig och/eller lider av panikattacker	☐	☐	☐	☐	☐
5. Jag blir störd av mycket folk och ljud	☐	☐	☐	☐	☐
Psykosomatiska besvär					
6. Jag lider av hjärtklappning eller bröstsmärtor	☐	☐	☐	☐	☐
7. Jag lider av mag- och/eller tarmbesvär	☐	☐	☐	☐	☐
8. Jag lider av huvudvärk	☐	☐	☐	☐	☐
9. Jag lider av muskelsmärtor, t.ex. i nacken, skuldrorna eller ryggen	☐	☐	☐	☐	☐
10. Jag blir ofta sjuk	☐	☐	☐	☐	☐

Citation: Schaufeli, W.B., De Witte, H. & Desart, S. (2019). *Burnout Assessment Tool (BAT) – Test Manual.* KU Leuven, Belgium: Internal report.
