# Supplementary material for: Burnout among midwives—the factorial structure of the burnout assessment tool and an assessment of burnout levels in a Swedish national sample
Source: BMC Health Serv Res. 2022 Sep 16;22:1167. doi: 10.1186/s12913-022-08552-8 (PMC9482233; doi:10.1186/s12913-022-08552-8)
Supplement: Supplementary file 2 — Additional file 2: Supplementary file 2. Frequency distribution of the item responses on the Burnout Assessment Tool in Swedish Midwives. [file 12913_2022_8552_MOESM2_ESM.pdf]

Supplementary file 2 – Frequency distribution of the item responses on the Burnout Assessment Tool in Swedish Midwives

Supplementary table 1 Percent (%) and count (n) of items responses on the Burnout Assessment Tool in Swedish Midwives (n=1664).

	Never	Rarely	Sometimes	Often	Always
**Item**	**% (n)**	**% (n)**	**% (n)**	**% (n)**	**% (n)**
EX1	13,1 (218)	34,7 (578)	34,6 (575)	16,5 (275)	1,1 (18)
EX2	5,2 (87)	23,9 (398)	39,9 (664)	26,6 (442)	4,4 (73)
EX3	9,5 (158)	32,1 (534)	37,3 (620)	17,4 (290)	3,7 (62)
EX4	22,7 (378)	39,8 (662)	28,7 (477)	8,2 (136)	0,7 (11)
EX5	12,7 (211)	35,7 (594)	34,3 (570)	14,4 (240)	2,9 (49)
EX6	32,9 (548)	43,1 (718)	18 (300)	5,2 (86)	0,7 (12)
EX7	24,5 (408)	37,3 (621)	25,2 (419)	11,2 (186)	1,8 (30)
EX8	7,6 (127)	28,7 (478)	36,1 (601)	22,7 (377)	4,9 (81)
MD1	22 (366)	43,7 (727)	24,9 (415)	7,9 (132)	1,4 (24)
MD2	38,8 (646)	38,6 (642)	17,4 (289)	4,8 (80)	0,4 (7)
MD3	63,5 (1057)	25,5 (425)	8,5 (141)	2,2 (37)	0,2 (4)
MD4	64,8 (1078)	23,9 (398)	8,7 (145)	2,5 (41)	0,1 (2)
MD5	69,4 (1155)	20,4 (340)	8,1 (134)	2 (34)	0,1 (1)
CI1	21,4 (356)	50,6 (842)	24,8 (413)	3 (50)	0,2 (3)
CI2	25,4 (422)	48,7 (811)	23,6 (392)	2,3 (38)	0,1 (1)
CI3	22,1 (367)	48,6 (809)	24,5 (407)	4,5 (75)	0,4 (6)
CI4	22,7 (378)	52,2 (868)	22,1 (367)	2,9 (49)	0,1 (2)
CI5	41,3 (687)	52,2 (869)	6,1 (102)	0,3 (5)	0,1 (1)
EI1	48,5 (807)	44,1 (733)	7 (117)	0,4 (6)	0,1 (1)
EI2	57,2 (951)	32,7 (544)	8,3 (138)	1,6 (26)	0,3 (5)
EI3	29,7 (494)	46,8 (779)	19,9 (331)	3,5 (58)	0,1 (2)
EI4	62,7 (1043)	28,2 (469)	8,1 (135)	1 (16)	0,1 (1)
EI5	55,1 (917)	36,2 (602)	7,8 (129)	0,8 (14)	0,1 (2)
