# Supplementary material for: Burnout among midwives—the factorial structure of the burnout assessment tool and an assessment of burnout levels in a Swedish national sample
Source: BMC Health Serv Res. 2022 Sep 16;22:1167. doi: 10.1186/s12913-022-08552-8 (PMC9482233; doi:10.1186/s12913-022-08552-8)
Supplement: Supplementary file 3 — Additional file 3: Supplementary file 3. Differential item functioning (DIF) for age. [file 12913_2022_8552_MOESM3_ESM.pdf]

Supplementary file 3 – Differential item functioning (DIF) for age

Supplementary table 2 Analysis of variance of residuals for test of DIF between age groups (under/above median age of 47) based on data from representative sample of Swedish midwives, subsample 1 n=800. Bonferroni adjusted significance level: 0.000145.

Item	MS	F	DF	Prob
EX1	8.37	10.25	1	0.0014
EX2	0.39	0.29	1	0.5890
EX3	1.05	1.16	1	0.2808
EX4	1.74	1.85	1	0.1738
EX5	1.66	1.90	1	0.1681
EX6	1.11	1.66	1	0.1973
EX7	1.01	1.19	1	0.2758
EX8	2.52	2.92	1	0.0881
MD1	2.03	2.33	1	0.1277
MD2	6.81	5.47	1	0.0196
MD3	0.69	0.73	1	0.3922
MD4	3.06	2.56	1	0.1100
MD5	0.05	0.03	1	0.8560
CI1	6.90	8.34	1	0.0040
CI2	3.58	4.64	1	0.0315
CI3	0.77	0.85	1	0.3561
CI4	0.89	1.12	1	0.2912
CI5	0.77	0.75	1	0.3878
EI1	2.54	2.46	1	0.1169
EI2	0.50	0.62	1	0.4318
EI3	0.10	0.09	1	0.7642
EI4	0.63	0.88	1	0.3491
EI5	13.11	11.78	1	0.0006
