# Supplementary material for: Burnout among midwives—the factorial structure of the burnout assessment tool and an assessment of burnout levels in a Swedish national sample
Source: BMC Health Serv Res. 2022 Sep 16;22:1167. doi: 10.1186/s12913-022-08552-8 (PMC9482233; doi:10.1186/s12913-022-08552-8)
Supplement: Supplementary file 4 — Additional file 4: Supplementary file 4. Observed residual correlation matrix. [file 12913_2022_8552_MOESM4_ESM.pdf]

Supplementary file 4 – Observed residual correlation matrix

Supplementary table 3 Observed residual correlation table for the Burnout Assessment Tool in Swedish midwives, subsample 1 n=800.

Item	EX1	EX2	EX3	EX4	EX5	EX6	EX7	EX8	MD1	MD2	MD3	MD4	MD5	CI1	CI2	CI3	CI4	CI5	EI1	EI2	EI3	EI4	EI5
**EX1**	1.0																						
**EX2**	.01	1.0																					
**EX3**	.09	.09	1.0																				
**EX4**	.13	.19	.23	1.0																			
**EX5**	.10	-.08	.25	.05	1.0																		
**EX6**	-.06	-.13	.03	.01	.12	1.0																	
**EX7**	-.02	-.05	.19	.13	.10	.21	1.0																
**EX8**	.22	.09	.28	.13	.17	-.03	.18	1.0															
**MD1**	.03	-.16	-.09	-.10	.19	-.01	-.05	-.03	1.0														
**MD2**	-.08	-.11	-.14	-.13	-.06	-.04	-.09	-.10	.14	1.0													
**MD3**	-.05	-.08	-.12	-.09	.03	-.13	-.14	-.11	.29	.01	1.0												
**MD4**	-.10	-.11	-.14	-.07	-.05	-.10	-.21	-.20	.16	.09	.29	1.0											
**MD5**	-.12	-.09	-.12	-.12	-.06	-.11	-.15	-.18	.13	.15	.09	.23	1.0										
**CI1**	-.09	-.17	-.19	-.23	-.15	.01	-.10	-.15	-.15	-.05	-.11	-.09	-.02	1.0									
**CI2**	-.15	-.16	-.17	-.15	-.16	.02	-.09	-.11	-.13	-.09	-.08	-.12	-.07	.48	1.0								
**CI3**	-.13	-.16	-.17	-.16	-.18	.00	-.08	-.15	-.20	-.08	-.14	-.13	-.13	.31	.36	1.0							
**CI4**	-.16	-.18	-.16	-.21	-.17	.01	-.09	-.18	-.16	-.15	-.13	-.11	-.10	.42	.47	.56	1.0						
**CI5**	-.15	-.08	-.25	-.21	-.22	-.05	-.16	-.28	-.15	-.05	-.07	-.06	-.07	.14	.17	.31	.30	1.0					
**EI1**	-.15	-.08	-.20	-.14	-.26	-.16	-.16	-.22	-.16	-.04	-.06	-.04	-.02	-.07	-.01	-.12	.00	.13	1.0				
**EI2**	-.12	-.13	-.18	-.19	-.20	-.05	-.17	-.19	-.13	-.07	-.05	.01	-.09	-.06	-.06	-.08	-.04	.04	.38	1.0			
**EI3**	-.15	-.08	-.16	-.16	-.17	-.21	-.16	-.21	-.17	-.12	-.04	-.05	.03	-.06	-.09	-.06	-.07	.04	.23	.22	1.0		
**EI4**	-.07	-.14	-.13	-.10	-.16	-.04	-.09	-.18	-.11	-.07	-.04	-.05	-.07	-.09	-.18	-.13	-.09	.01	.23	.32	.20	1.0	

**EI5**	-.13	-.08	-.21	-.14	-.25	-.13	-.16	-.15	-.14	.02	-.09	-.04	-.02	-.14	-.17	-.11	-.19	.06	.34	.24	.33	.34	1.0
